# Help-seeking behaviour for depressive disorders among adult cardiovascular outpatient cardiac clinic Jimma University Teaching Hospital, Jimma, South-West Ethiopia: crosssectional study

**DOI:** 10.1186/s13033-019-0262-2

**Published:** 2019-01-31

**Authors:** Asmare Belete, Alemayehu Negash, Mengesha Birkie

**Affiliations:** 1 Department of Psychiatry, College of Medicine and Health Sciences, Wollo University, Dessie, Ethiopia; 2 Department of Psychiatry, College of Public Health and Medical Sciences, Jimma University, Jimma, Ethiopia

**Keywords:** Cardiovascular disease, Depression, Help seeking behavior, Determinant of help seeking, Ethiopia

## Abstract

**Background:**

Depression in healthy person without cardiac disease has been associated with the development of coronary artery disease and cardiovascular disease also risk factor for development of depression. This has devastating effect the patient’s quality of live, illness progression, morbidity and mortality. Despite this fact help seeking behavior of cardiovascular patients with depression has not been addressed in Ethiopia.

**Objective:**

To assess help-seeking behaviors of adult cardiovascular patients with depression for their depressive disorders in Jimma university teaching hospital.

**Method:**

Institution based cross sectional study conducted October to December in 2014. The study was conducted on 353 cardiovascular patients who attended at cardiac clinic. Depression was assessed using patient health questionnaire version nine (PHQ-9), which is validated in Ethiopia, Help seeking behavior using actual help seeking questionnaire and social support using Oslo social support-3 item scale.

**Result:**

From the total of 339 participants, 57.5% (n = 195) of them fulfill the case definition of depression and 12.1% (n = 41) of participant reported idea of hurting themselves. Only 33.3% sought help for their depression. Of those participants who sought help, 88.6% sought help from one or more of an informal help source. Occupation (odds of = 4.24, 95% confidence interval (CI) 1. 31, 13.78), education level (AOR 7.6, CI 2. 13, 27.11), the presence of a history of mental illness in the family (AOR 7.33, CI 2. 72, 19.80), ideal of hurting themselves, knowing the availability of the psychiatric service in this hospital and having previous seeking help were significantly associated with help seeking behavior.

**Conclusion and recommendation:**

The number of patients not seeking help for depression is high. There for scaling up mental health service in tertiary hospitals through multidisciplinary approach should be given high priority.

## Background

According to World Health Organization (WHO) mental health is defined as a state of subjective well-being in which an individual realizes his or her own abilities, can cope with the normal stresses of daily life events, can work productively and able to make a contribution to his or her society [1]. Depression is a serious mental illness that affects one’s thoughts, feelings, behavior, mood and physical health. Depression is a life-long condition in which periods of wellness interchange with recurrences of illness [2]. Co-morbid depression is the existence of a depressive disorder (i.e. major depression, dysthymic or adjustment disorder) along with a physical disease [3]. Those co-occurrence of diseases increased patients’ risk of disability and mortality [4]. But in the other report of this organization illustrate that in worldwide thousands of people with mental illness did not get mental health Services [5].

Fifty-seven million deaths occurred in the world during 2008; out of this (63%) were due to NCDs. Almost 80% of these NCD deaths occurred in LMIC [6]. Chronic non-communicable cardiovascular diseases are the leading cause of death in the world [3] and also rapidly overtaking infectious diseases as the major cause of death and disability in the developing world [7].

Depression is one of the leading contributors of the burden of disease globally and in low- and (LMIC), and is projected to be, overall, the second leading cause of burden of disease by 2020 [8, 9].

Major depression disorder (23.8%) and sub-syndrome symptom of depression (20.8%) is highly prevalent among Myocardial Infraction patients. But depression among this groups of patients remain unrecognized and untreated [10].

The syndrome of major depression is present in approximately 15% of patients with cardiac disease; such a rate is substantially higher than that seen in the general population (4% to 5%) Or primary care patients (8% to 10%). And also in other study depression in healthy persons without cardiac disease has been associated with the development of coronary artery disease; it associated with a 60% increase in cardiac disease [11–14]. Depression is an independent risk factor for the development of CAD. Patients with CAD have a high rate of depression, which worsens their prognosis [15].

Depression among hypertensive patients is also highly prevalent; it’s also not only chronicity of hypertension increase depression prevalence, instead pathophysiological bidirectional related. Comorbidity of depression and hypertension fasten disease progression to cardiac complication [16]. In our country also NCD are the leading contributor of (51%) death among adults in Addis Ababa, where the health care system is still gives great attention toward addressing communicable diseases [17].

## Help seeking tendency

A study done in New York in 2012, a majority (61.3%) of respondents with lifetime major depression disorder (MDD) (N = 5, 958) reported having help seeking for depression treatment [18].

A study done among African Americans for screening depression using the International Diagnostic Inventory, out of 441 participants, 66.4% were classified as affective depression, 17.8% complicated depression, and 15.8% as physical depression. From these groups, complicated depression group was associated with increased likelihood seeking treatment from a mental health professional. Seeking treatment from a family doctor was associated with physical depression. Seeking care from three or more different health care providers was associated with complicated depression [19].

Community based screening study done in Butajira, Ethiopia 2009, indicated that over half of the cases (55.9%) had never sought help from the modern health care sector, and only 13.2% had ever been admitted to psychiatric hospital [20]. These data suggest that pharmacological and non- pharmacological treatment of depression might improve the quality of life (QOL) of heart failure (HF) patients [21]. Thus heart failure patients who get treatment for their depression, quality of life will improve.

Study done in Italy among 18–69 years old revealed that 34% had sought help from a health professional, 13% from family or friends, and 6% from both. The remaining 47.2% had no sought help. Factors significantly associated with not having helped sought from any (either) source were male sex, being regularly employed and age 18–34 years old [22]. Study done in the Meskan and Mareko district in Ethiopia among general population only 33.4% of respondents with persistence depression sought help from any kind in the 3 months follow up assessment. Out of respondents with persistence depression; 16.7% use government primary health care service, 9.3% private healthcare and 7.4% traditional and religious healers [23].

Overall depression is a major public health problem worldwide; but its’ burden increased while it co-occur with chronic medical illness like cardiovascular. The prevalence of depression become alarmingly increasing with patients who have chronic co-morbid medical illnesses such as cardiovascular disease. Patients with depression do not seek-help, even if it has a great negative impact on quality of life, productivity, social functioning and accelerating chronic disease prognosis it still remains undetected and under treated.

## Methods and materials

### Study area and period

The study was conducted in JUTH is located 352 km south west of the capital city from Ethiopia, Addis Ababa. Jimma University Tertiary Teaching Hospital is one of the oldest public hospitals in the country. It was established 1937 during Italian occupation to give service for their soldiers. It provides services for approximately 9000 inpatient and 80,000 outpatient attendances a year coming to the hospital from the catchment population of about 15 million people.

Cardiac Clinic is one of the follow-up clinics giving service for patient with chronic CVDs among others clinics that give service for patients with other chronic NCDs. This clinic gives service for a total of 1939 adult cardiac patient for follow up their cardiac status and to take medication. Data were collected from adult patient from October to December 2014.

### Participants

The study participants were all adult patients who had cardiovascular diseases age 18 years and above who came for follow-up at JUTH cardiac clinic during the study period. A total of 353adult patients who had cardiovascular diseases and Age 18 years or older were involved in the study. Systematic random sampling method was employed. This study exclude patients having hearing problem and severe mental illness except depression but patients with depressive disorder presented in psychomotor retardation or catatonic features were excluded.

### Measurements

The dependent variable was help seeking behavior. The independent variables includes socio- demographic characteristics such as age sex, religion, ethnicity, educational status, occupation, residency, marital status and also psycho-social related factors and illness-related factors.

### Data collection procedures and instruments

A structured interviewer administer questionnaire was used. Depression was measured using Patient Health Questionnaire nine (PHQ-9) which is a validated instrument in Ethiopia [24]. For help seeking behavior, we used the Actual Help Seeking Questionnaire designed and used for the assessment of recent help seeking of patients with CVD for emotional problems for the last 2 weeks just prior to the date of being interviewed [25]. Pre-test was conducted on 5% of the sample size before the main study was done. Amharic and Oromifa version of questionnaire were used for data collection.

### Data collectors’ selection and training

Data were collected by six BSc nurses. Supervision was made by one Masters in Public Health and principal investigator. Data collectors and supervisor were trained for 1 day by the principal investigator on the study instrument, consent form, how to maintain confidentiality and data collection procedure based on AHSQ.

### Data quality management

One day training of data collectors was given on how to collect data. Regular supervision by the supervisor and the principal investigator was made to ensure that all necessary data were properly collected. Each day during data collection, filled questioners were cheeked for completeness and consistency. Questionnaire which was not completely filled it was discarded.

### Data processing and analysis

The quantitative data was entered into the computer by using Epi-data version 3.1 and lastly exported to SPSS version 21 for analysis. The data was explored by using frequency tables and figure. Measure of central tendency was calculated and utilized for appropriate variable to describe, the data, to check for consistencies and to identify missed values. Bivariate analysis and multiple logistic regressions were used. Finally, variables had *p* value of less than 0.25 on binary logistic regression were entered into multivariable logistic regression. Then, variables which showed statistical significant association with p-value less than 0.05 on final model were considered as predictors of help seeking behaviors.

### Ethical considerations

The ethical approval was received from the institutional review board of Jimma University College of Public Health and Medical Sciences. Written informed consent was obtained from the Study participants. The data given by the participants was used only for research purposes. Participants have the right to late the participation.

## Results

### Socio-demographic characteristics of study subjects

From the total of 353 cardiovascular patients 339 of them completed the questionnaire with a response rate of 96%. Among the 339 respondents 53.1% (n = 180) were females making female to male ratio of 1.13:1. The mean age of the study participants was 50.1 (SD ± 17.11; median 51.22) year. Among the respondents, Oromo ethnic group constituted 77.3% (n = 262). Majority of the study population were married (76.4%). In terms of residence, rural study participants surrounding Jimma Town constituted the majority (64.0%). Concerning religion of participants, Islam constituted a great majority (75.2%). With regards to occupation, out of the study population more than half of them were farmers (50.7%). The median annual income of the participants, as reported by them, was 3000.00 (mean, 7,862.94) ETB (Table 1).

**Table 1:** Socio-demographic characteristics of the study participants and association with seeking any form of help, Jimma University Teaching hospital, Ethiopia December 2014

Factors	Frequency	
	Number (n = 339)	Percent
Sex		
Male	159	46.9
Female	180	53.1
Age of the respondent		
18–27	45	13.3
28–37	48	14.2
38–47	55	16.2
48–57	55	16.2
58–67	81	23.9
≥ 68	55	16.2
Occupation		
Farmer	172	50.7
Unemployed	67	19.8
Housewife	29	8.6
Merchant	23	6.8
Employed	18	5.3
Daily laborer	8	2.4
Retired	13	3.8
Others^a^	9	2.7
Income of the respondent (Birr)		
< 900	84	24.8
900–2999	75	22.1
3000–9999	85	25.1
≥ 10,000	95	28.0
Marital status		
Married	259	76.4
Others^b^	80	23.6
Oromo	262	77.3
Ethnicity		
Amhara	37	10.9
Yem	16	4.7
Gurage	8	2.4
Others^c^	16	4.8
Religion		
Muslim	255	75.2
Orthodox	71	20.9
Protestant	13	3.8
Attending place of worship		
Daily	119	35.1
2–3 times per week	45	13.3
Once per week	150	44.2
Less than a week	25	7.3
Illiterate	180	53.1
Educational level		
Able to read and write only	68	20.1
Formal education	91	26.8

Others^a^ = student, house servants others^b^ = single, divorced separated others^c^ = Tigra, Dawero, Welayeta and Kefa

### Illness related characteristic of cardiovascular patient outpatient cardiac clinic

Out of the total of 339 CVD patients, 7.1% (n = 24) reported past history of thought of hurting themselves and also 12.1% (n = 41) of participants reported having current thinking of hurting themselves within the study period. When we see the comorbid illness, nearly half of patients reported one or more comorbid medical health problem in addition to CVD. Out of depressive CVD patient who had previous consultation for their depression was 15.4% (n = 30) sought help for their depression. Regarding the diagnosis; majority of them (34.8%) had hypertensive related heart disease. Followed by 28.0% (n = 95) had ischemic heart disease, myocardial infarction, and Acute coronary syndrome. With regard to duration of CVD of the respondents; around 26% of participants had 1–3 years (Table 2).

**Table 2:** Illness related characteristic of cardiovascular patients in outpatient cardiac clinic JUTH south west Ethiopia, December 2014

Factors	Frequency (n = 33 9)	Percent
History of suicidal thought		
Yes	24	7.1
No	315	92.9
Suicidal ideation		
Yes	41	12.1
No	298	87.9
Comorbidity other than heart disease		
Yes	168	49.6
No	174	50.4
Diagnosis		
HHD	118	34.8
IHD^a^	95	28.0
Cardiomyopathy	48	14.2
VHD/RF	34	10.0
DHD	29	8.6
Cor-plumonary	10	2.9
Others^b^	5	1.5
Duration of CVD disease		
< 1 year	82	24.2
1–3 years	90	26.5
4–5 years	70	20.6
6–7 years	44	13.0
≥ 7 years	53	15.6

HHD—hypertensive related heart disease, VHD—vulvular heart diseases, and RF—heart disease due to rheumatic fever and DHD—diabetic related heart disease

^a^ Ischemic heart disease (IHD), acute coronary syndrome, myocardial infarction and angina

Others^b^ arrhythmia and thyrotoxicosis

**Fig. 1:**
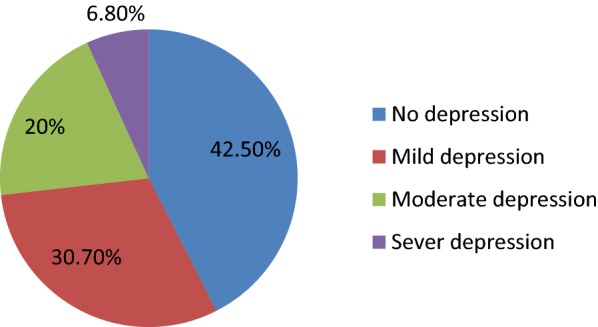
Severity of depression of CVD patients’ at cardiac clinic JUTH southwest Ethiopia December 2014

Regarding severity of depression; according to PHQ-9, 42.5% (n = 144) had no depression; 30.7% (n = 104) had mild depression, 20.0% (n = 68) of them moderate depression. Participants with severe depression were 6.8% (n = 23) severe depression (Fig. 1).

**Fig. 2:**
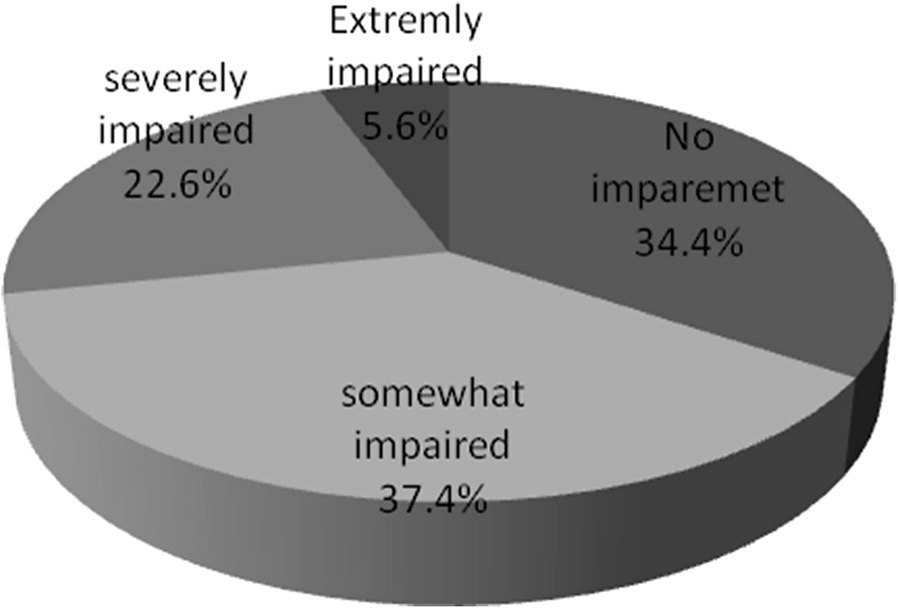
Functionality of CVD patients with depression in cardiac clinic JUTH, December 2014

Based on the patients’ report on functionality, 37.4% (n = 73) where somewhat impaired whereas 22.6% (n = 44) were severely impaired and 5.6% (n = 11) reported extreme impairment to accomplish their day to day activities because of the depressive symptoms for the last 2 weeks prior to data collection period. Even if patients had sign and symptom of depression, 34.4% (n = 67) reported their functionality was intact (Fig. 2).

### Psycho-social and behavioral factors

Among the total sample of cardiovascular patients, (38.9%) participants reported poor social support, 38.3% moderate support and the rest (22.2%) strong social support. Concerning information about mental illness, 63.7% (n = 216) had heard about mental illness. From the total of 339 respondents 44.4% (n = 152) CVD patients believed life stressors alone as a cause for mental illness (Table 3).

**Table 3:** Psycho-social and behavioral factors of cardiovascular patient outpatient cardiac clinic JUTH, southwest Ethiopia, December 2014

Factors	Frequency (n = 339)	Percent
Living condition		
With family	304	89.7
Live alone	24	7.1
Other^a^	11	3.3
Social support		
Poor	132	38.9
Moderate	130	38.3
Good	77	22.7
Information about MI		
Yes	216	63.7
No	123	36.3
Neighborhood	92	42.6
MI information source		
From religious leaders	33	15.3
From mass media	91	42.1
Presence of other mental illness in the family		
Yes	65	19.2
No	274	80.8
Awareness of MH service availability Hospital		
Yes	204	60.2
No	135	39.8
Cause of MI		
Evil or bad sprit	37	10.90%
Stress life events	152	44.80%
Genetic predisposition	42	10.90%
More than one of the above	108	31.90%
Fear stigma from the public		
Yes	22	11.3
No	173	88.7
Life time cigrate use		
Yes	30	8.8
No	309	91.2
Current cigrate use		
No	325	95.9
Yes	14	4.1
Life time alcohol use		
Yes	41	12.1
No	298	87.9
Current alcohol use		
No	320	94.4
Yes	19	5.6

Other ^a^—live with relative, homeless or living in employers ‘home

### Prevalence of help seeking behavior for depression among cardiovascular patients

#### Help seeking behavior associated with socio-demographic factors

Using Actual Help Seeking Behavior Questionnaire (AHSQ), 33.3% 95%CI (26.69, 39.91) (n = 65) of depressed cardiovascular sought help for their depression in the last 2 weeks. But majority of respondents did not seek help from any form of help source (66.7%). Significant portion of females did not seek help for their depression (77.7%; n = 78). Nearly half of the participants with age group 58–67 sought help. Out of respondents with depression who were in the age group greater than or equal to 68, 40.0% (n = 14) of them sought help for depression from any form of help sources. Majority of single, divorced and windowed patients never sought help (75%, n = 37).

Those who were able to read and write 76.1% (n = 35) as well as 72.5% (n = 37) of the illiterates never sought help. Out of CVD patient with depression who had annual income less than 900.00 Ethiopian Birr, 67.2% (n = 72) did not sought help for their depression. Finally, from demographic part, residence is the factor that have implication on help sought among depressive CVD patients; so 67.7% (n = 84) patient who live from rural part of Jimma Town never sought (Table 4).

**Table 4:** Distribution of socio-demographic factors of actual help seeking behavior for depression by socio-demographic characteristics, Jimma University, Ethiopia, November, 2014

Factors	Help seeking		COR (95%CI)	P-value
	Yes	No		
Sex				
Male	42 (44.7%)	52 (55.3%)	2.74 (1.47–5.08)	0.001
Female	23 (22.8%)	78 (77.2%)	Ref	
Age of respondent				
18–27	5 (20.8%)	19 (79.2%)	0.32 (0.1–0.99)	0.49
28–37	7 (20.6%)	27 (79.4%)	0.31 (0.11–0.86)	0.025
38–47	10 (40.0%)	15 (60.0%)	0.8 (0.0.29–2.16)	0.66
48–57	9 (27.3%)	24 (72.2%)	0.45 (0.17–1.18)	0.1
58–67	20 (45.5%)	24 (54.5%)	Ref	
≥ 68	14 (40.0%)	21 (60.0%)	0.8 (0.32–1.96)	0.63
Marital status				
Married	53 (36.3%)	93 (63.7%)	Ref	
Others^a^	12 (24.5%)	37 (75.5%)	0.57 (0.27–1.18)	0.13
Ethnicity				
Oromo	53 (34.6%)	100 (65.4%)	Ref	
Amhara	5 (26.3%)	14 (73.7%)	0.67 (0.23–1.97)	0.47
Others^b^	7 (30.4%)	16 (69.6%)	0.82 (0.32–2.13)	0.69
Religion				
Muslim	53 (34.6%)	100 (65.4%)	Ref	
Christian	12 (28.6%)	30 (71.4%)	0.75 (0.36–1.59)	0.46
Attending place of worship				
Daily	24 (33.8%)	47 (66.2%)	1.33 (0.67–2.65)	0.41
2–3 times per week	11 (42.3%)	15 (57.7%)	1.91 (0.77–4.77)	0.16
Once per week	23 (27.7%)	60 (72.3%)	Ref	
Less than per week	7 (46.7%)	8 (53.3%)	2.28 (0.74–7.01)	0.15
Educational level				
Illiterate	35 (32.7%)	72 (67.3%)	1.55 (0.70–3.40)	0.27
Able to read and write only	11 (23.9%)	35 (76.1%)	Ref	
Formal education	19 (45.2%)	23 (54.8%)	2.63 (1.10–6.53)	0.037
Annual income of respondent (Birr)				
Less than 900	14 (27.5%)	37 (72.5%)	Ref	
900–2999	12 (27.9%)	31 (72.1%)	1.02 (0.413–2.53)	0.96
3000–9999	16 (32.0%)	34 (68.0%)	1.24 (0.53–2.93)	0.61
≥ 10,000	23 (45.1%)	28 (54.9%)	2.17 (0.95–4.96)	0.06
Occupation				
Unemployed	10 (18.2%)	45 (81.8%)	Ref	
Employed	7 (38.9%)	11 (61.1%)	2.86 (0.89–9.22)	0.07
Farmer	41 (42.7%)	55 (57.3%)	3.35 (1.51–7.43)	0.003
Others^c^	7 (26.9%)	19 (73.1%)	1.66 (0.55–5.00)	0.37
Residence				
Rural	40 (32.2%	84 (67.7%)	Ref	0.67
Urban	25 (35.2%)	46 (64.8%)	1.14 (0.62–2.11)	

^a^ Single, windowed/divorced

^b^ Yem, Tigra, Dawero, Gurage, welayeta and/kefa

^c^ In occupation who are house wife, student and retire

#### Help sought for depression associated with illness-related, psycho-social and behavioral factors

##### Illness related factors

Among study population who had suicidal thought half of them had visited one or more help sources. Regarding severity of depression, only 27.9% (n = 29) of mild depression sought help from any source. Out of CVD patients with depression who reported having of extremely functional impairment, 54.5% (n = 6) sought help for their depression. Out of those who had previous consultation for their depression nearly two-third of them currently also sought help (Table 5).

**Table 5:** Distribution of help seeking behavior for depressive disorders in related to illness related factors of CVD patients JUTH, Jimma South west Ethiopia, 2014

Factors	Help seeking		COR (95%CI)	p-value
	Yes	No		
History of suicidal attempt				
Yes	8 (42.1%)	11 (57.9%)	Ref	
No	57 (32.4%)	119 (67.6%)	0.66 (0.25–1.73)	0.34
Suicidal ideation				
Yes	19 (51.4%)	18 (48.6%)	2.57 (1.24–5.33)	
No	46 (29.1%)	112 (70.9%)	Ref	0.01
Co morbidity medical illness other than heart disease				
Yes	31 (31.9%)	66 (68.1%)	Ref	
No	34 (34.7%)	64 (65.3%)	1.13 (0.62–2.05)	0.68
Duration of CVD illness				
< 1 year	12 (27.9%)	31 (72.1%)	0.57 (0.24–1.35)	0.20
1–3 years	21 (40.4%)	31 (59.4%)	Ref	
3–5 years	16 (39.0%)	25 (61.0%)	0.94 (0.41–2.18)	0.89
5–7 years	9 (32.1%)	19 (67.9%)	0.47 (0.26–1.84)	0.47
> 7 years	7 (22.6%)	24 (77.4%)	0.43 (0.16–1.18)	0.10
Severity of depression				
Mild	29 (27.9%)	75 (72.1%)	Ref	
Moderate	25 (36.8%)	43 (63.2%)	1.50 (0.78–2.89)	0.22
Sever	11 (47.8%)	12 (52.2%)	0.06 (2.37–0.94)	0.06
Functionality impairment				
No difficulty	18 (25.0%)	54 (75.0%)	Ref	
Somewhat difficult	20 (29.0%)	49 (71.0%)	1.22 (0.58–2.58)	0.6
Very difficult	21 (48.8%)	22 (51.2%)	2.86 (1.28–6.38)	0.01
Extremely difficult	6 (54.5%)	5 (45.5%)	3.6 (0.98–13.22)	0.05
Previous consultation				
Yes	30 (66.7%)	15 (33.3%)	Ref	
No	35 (23.3%)	115 (76.7%)	0.15 (0.07–0.32)	0.001

##### Psycho-social and behavioral factors

Concerning living condition, out of depressive cardiovascular patients who live with his family 58.5% (n = 114) did not sought help for their depression. Regarding social support, those participants with depression who have strong social support nearly half (46.8%, n = 22) of them sought help for their depression. While those one with poor social support only 26.1% sought help for their depression. Out of respondents with depression who had no information about mental illness 74.7% (n = 59) never sought help for their depression. Out of participants with depression who had presence of mental ill patients in the family members 65.1% sought help. Among depressed cardiovascular patients who believe cause of mental illness was from genetic predisposition only 25.0%, 35.0% evil or bad sprit, 37.0% more than one of the mentioned causes ware sought help their depression (Table 6).

**Table 6:** Distribution of help seeking behavior for depression disorders in related to behavioral and psycho-social factors of CVD patients JUTH, Jimma December 2014

Factors	Help seeking		COR (95%CI)	p-value
	Yes	No		
Living condition				
With family	60 (34.5%)	114 (65.5%)	Ref	
Others^a^	5 (23.8%)	16 (76.2%)	1.68 (0.59–4.80)	0.33
Social support				
Poor	23 (26.4%)	64 (73.6%)	2.45 (1.16–5.16)	0.019
Moderate	20 (32.8%)	41 (67.2%)	1.80 (0.82–3.95)	0.14
Strong	22 (46.8%)	25 (53.2%)	Ref	
Information mental illness				
Yes	45 (38.8%)	71 (61.2%)	Ref	
No	20 (25.3%)	59 (74.7%)	1.87 (1.02–3.51)	0.051
Source of information about mental illness				
Neighborhood	22 (38.6%)	35 (61.4%)	0.88 (0.32–1.97)	0.75
Religious leader	7 (41.2%)	10 (58.8%)	0.79 (0.25–2.47)	0.68
Mass media	16 (35.6%)	29 (64.4)	Ref	
I did not hear information	20 (26.3%)	56 (73.7%)	1.54 (0.69–3.42)	0.28
Presence of mental illness in the family				
Yes	28 (65.1%)	15 (34.9%)	Ref	0.001
No	37 (24.3%)	115 (75.7%)	5.8 (2.8–12.02)	
Availability of MI service in this hospital				
Yes	43 (39.4%)	66 (60.6%)	Ref	0.04
No	22 (25.6%)	64 (74.4%)	2.0 (1.02–3.52)	
Believe of respondent about Case of MI				
Bad/evil sprit	6 (35.3%)	11 (64.7%)	1.10 (0.36–3.32)	0.87
Stress	27 (31.4%)	59 (68.8%)	1.31 (0.68–2.52)	0.42
Genetic predisposition	5 (25.0%)	15 (75.0%)	1.80 (0.58–5.51)	0.3
More than one of the above	27 (37.5%)	45 (62.5%)	Ref	
Life time cigarette use				
Yes	10 (47.6%)	11 (52.4%)	0.58 (0.20–1.27)	0.15
No	55 (31.6%)	119 (68.4%)	Ref	
Current cigarette use				
No	60 (32.8%)	124 (67.2)	Ref	
Yes	5 (41.7%)	6 (58.3%)	0.58 (0.17–1.98)	0.38
Life time alcohol use				
Yes	9 (47.4%)	10 (52.6%)	0.52 (0.20–2.34)	0.48
No	56 (31.8%)	120 (66.7%)	Ref	
Current alcohol use				
No	60 (32.8%)	123 (67.2%)	Ref	
Yes	5 (41.7%)	7 (58.3%)	0.68 (0.21–2.24)	0.53
Life time khat use				
Yes	24 (43.6%)	31 (56.4%)	0.54 (0.21–1.02)	0.05
No	41 (29.3%)	99 (70.7%)	Ref	
Current khat use				
No	60 (33.1%)	121 (66.9%)	Ref	
Yes	5 (35.7%)	9 (64.3%)	0.89 (0.29–2.78)	0.85

Others^a^ -living alone, live with relative and homeless

### Pattern of help seeking of depressed cardiovascular patients

Among depressed cardiovascular patients which account 66.7% (n = 130) did not sought help for their depression. Among help source visited by patients; the most frequently visited help was informal help source (88.6%; n = 156). In contrast to this, only 11.4% (n = 20) had sought help from formal source of help for their depression (Table 7).

**Table 7:** Help Sources with depressed cardiovascular patients actually seek help on the past 2 week for their depression, Jimma University, Ethiopia, December 2014

Help source	Frequency	%
Informal help source		
Traditional healer	47	27.3%
Relatives	25	14.2%
Husband/wife/intimate partner	30	17.0%
Minister/religious leader	30	17.0%
Neighbor	13	7.4%
Parent	10	5.7%
Total	156	88.6%
Formal help source		
Mental health professional	3	1.8%
Doctor/GP or other health	17	9.6%
Professional	20	11.4%
Total		

The total number of help sought greater than sample of patients (65) who had sought help for their depression because of multiple responses given by the participants

### Associated factors with seeking any form of help

#### Factors that associated with help seeking behavior for depression in first model analysis among depressive case of cardiovascular patient JUTH

Out of different groups of variables marital status, frequency of attending place of worship, annual income of the respondent, Severity of depression, history of life time chat use, information about mental illness, duration of CVD illness, history of life time alcohol use, history of life time cigarette use were associated with help seeking behavior of CVD patients for their depression (p < 0.25).

Other variables such as male, age, able to read and write, unemployed, poor social support, presence of mental illness in the family, awareness of availability of psychiatric service in JUTH, current suicidal thought, burden of depression that affect his life, and previous consultation were associated with help sought in binary logistic regression analysis at p-value < 0.05 (Tables 4, 5 and 6).

#### Factors that associated with help seeking behavior for depression in final model

Variable which had independent significant association with help sought for depression in the final model were female AOR 1.46 (0.39–5.40), being farmer AOR 4.24 (1.3, 13.78; p = 0.007), formal education AOR = 7.59 (2.13–27.11); p = 0.002), had family history of mental illness AOR 7.33 (2.72–19.78; p < 0.001), had awareness of the availability of psychiatric service in this hospital AOR 3.54 (1.41–8.92; p = 0.012), current suicidal ideation AOR 4.0 (1.33–12.03; p = 0.013), had very difficult of impairment in functionality AOR = 4.98 (1.50–16.50.) and lastly, cardiovascular patients who had no previous history of seeking help for their depression were 87% less likely to sought help for their depression than those who had previous history of consultation, AOR 0.13 (0.04–0.34; p < 0.001) (Table 8).

**Table 8:** Multivariate logistic regression of factors associated with help seeking behavior for depression among cardiovascular patient with current depression JUTH, Jimma Southwest Ethiopia December 2014

Factors	Help seeking		COR (95% CI)	AOR (95% CI)
	yes	No		
Occupation				
Unemployed	10 (18.2%)	45 (81.8%)	Ref	Ref
Employed	7 (38.9%)	11 (61.1%)	2.86 (0.89–9.22)	2.07 (0.39–10.87)
Farmer	41 (42.7%)	55 (57.3%)	3.35 (1.51–7.43)	4.24 (1.30–13.78)
Others^a^	7 (26.9%)	19 (73.1%)	1.66 (0.55–5.00)	0.40 (0.08–1.96)
Educational level				
Illiterate	35 (32.7%)	72 (67.3%)	2.52 (0.84–7.53)	2.52 (0.84–7.53)
Read and write only	11 (23.9%)	35 (76.1%)	Ref	Ref
Formal education	19 (45.2%)	23 (54.8%)	7.59 (2.13–27.11)	7.59 (2.13–27.11)
MI in the family				
Yes	28 (65.1%)	15 (34.9%)	5.8 (2.8–12.02)	7.33 (2.72–19.8)
No	37 (24.3%)	115 (75.7)	Ref	Ref
Awareness of MI service in this hospital				
Yes	43 (39.4%)	66 (60.6%)	1.89 (1.02–3.51)	3.15 (1.3–7.69)
No	22 (25.6%)	64 (74.4%)	Ref	Ref
Suicidal ideation				
Yes	19 (51.4%)	18 (48.6%)	2.57 (1.23–5.33	4.0 (1.33–12.03)
No	46 (29.1%)	112 (70.9%	Ref)	Ref
Distress felt by patients				
No difficulty	18 (25.0%)	54 (75.0%)	Ref	Ref
Somewhat difficult	20 (29.0%)	49 (71.0%)	1.22 (0.58–2.58)	1.45 (0.55–3.85)
Very difficult	21 (48.8%)	22 (51.2%)	2.86 (1.28–6.38)	4.98 (1.50–16.50)
Extremely difficult	6 (54.5%)	5 (45.5%)	3.6 (0.98–13.22)	2.99 (0.36–24.90)
Previous consultation				
Yes	30 (66.7%)	15 (33.3%)	Ref	Ref
No	35 (23.3%)	115 (76.7)	0.15 (0.07–0.32)	0.13 (0.04–0.34)

Others ^a^ in occupation who are house wife, student, retire, house servant

## Discussion

This is the first of its kind study on help seeking behavior of adult CVD patients with depression in Ethiopia and perhaps in sub-Saharan Africa to my knowledge. The finding that more than two-third of the total CVD depressed patients did not seek help which is very high. It needs due attention of policy makers, health service program designers and team approach from different specialty clinical of discipline. Because of this it was not possible to compare results with those studies conducted on help seeking behavior of patients with other health problems. However comparing this result, with other study might be indicative of the awareness and magnitude of CVD patients suffering from comorbid depression compared to other patients’ help seeking behavior. From Cardiovascular patients with comorbid depressive disorders, only one-third of participants were found to seek help for their depression from any form of help sources. This could be explained by that CVD patients with depression might not be aware of that depression is treatable, may perceive their feeling result of CVD or those who have awareness might not seek help in mental health setup fear of stigma. This result is higher than study done in Ethiopia [26]. Firstly, the reason might be presence of chronic co morbid medical illness. Patients with comorbidity more likely to seek help for their depression than those did not have comorbid illness [27]. Secondly, this might be due to that the last study took in consideration only individuals that sought help from psychiatrist. But our study includes utilization of other source like mental health professional, counselor, general practitioner, health officer, other health professionals and informal help sources. Similarly, patients in this study had contact with health professional and might get advice from treating health professionals to seek help for their emotional problem. Type of help sources used by the participants for their depression could be the other reasons that contribute for large number of patients sought help in this study. But it is lower than studies conducted in developed countries like from New York (61.3%), Italy (52.8%) and South London (66.7%) [18, 22, 23]. Possible explanation for the difference might be knowledge gab about depression, clinician working at cardiac clinic douse not identify/pay attention for depression and consult.

Our study help sought from formal source is very low as compared to other studies. This shows that patient who sought help for depressive disorder from psychiatrist, mental health professional, and psychologist and even general practitioner and other health professional is minimal. But the prevalence of depression among thus study population is high. The possible reason could be treating physician did not pay attention to screen for comer bid depression.

In our study, educational level of patients with depression is one of the independent predictor of help seeking for depression. Accordingly, CVD patients with depression who had some formal education were 7.6 times increased odds of seeking help as compared to those able to read and write only. But it was in similar with the study done from Ethiopia who reported patients with educational level 5–12 grade have greater odds of visiting health facility than illiterate [26]. This is contradicting with the study done in Norway, among adult with anxiety disorder and depression [28]. Firstly, possible reason might be socio cultural difference. Study done in psychiatric clinic of this hospital on pattern of treatment seeking behavior for mental illness in 2011 depict that presence of other family members with mental illness associated with increased likelihood of help sought for their mental disorder [29]. Our finding also similar with the above mentioned study.

Socio-economic status of the patients could be one of the factors that determine their help sought for their emotional problem. The individual with full time or par time worker 1.4 times odds of seeking help for their depression than who did not work [22]. Similarly, in this paper also, being farmers 4.24 increased odds of help sought for their depression than unemployed. The possible reason is that most of our participants seek help from informal source of help; Severity of their depression could be the other possible reasons that enforce them to sought help for severe emotional problem.

Those participants who have awareness availability of psychiatric service in this hospital 3.5 times increased odds of help seeking for their depression than those participants that have no awareness availability of the service. This result is unique for this study and it may consider as new finding. Possible reason could be patient with depression who aware mental illness is treated in this hospital; might aware that depression is one of mental illness that can be treated here. Qualitative study done in United Kingdom; among coronary heart disease or diabetic patients to assess believe about depression. Depressed patients were unsure to seek help for their depression from others even they had suicidal ideation. In the same study, depression free patient believe that suicide is only consider seeking help for depression [30].

In our study CVD patients with depression who had current suicidal ideation has increased chance of seeking help for their depression as compared to those has no suicidal ideation. This could be because of patients with suicidal ideation were severely impaired that might enforce them to seek help. Out of CVD patient with depression, who had no past history of seeking help for their depression is 87% less likely to seek help than those participants with past history of consultation. These patients with previous consultation had increased chance of to seek help for current depression as well. The possible reason could be they were might satisfied on previous consultation, and again they use.

In this study, CVD patient with depression functional impairment independent predictors of help seeking behavior for depression. As a result, it was very difficult to perform their day to day activity three times increased odds of seeking help for their depression as compared to no difficulty. This could be due to nature of depression itself. In general, the more severity of depression the greater chance of a person impaired to perform their day to day function. So they tried to seek help for depression in order to accomplish their day to day activities.

## Conclusion and recommendation

### Conclusion

The result showed alarmingly high numbers of these patients have not sought any kind of help for their depression. Factors found to be significantly associated with help seeking behavior include occupation, suicidal ideation, educational level, presence of other family members with mental illness, previous consultation to their depression, awareness about availability of mental health service in this hospital and functional impairment due to depression. This result shows that intervention are needed to improve help seeking tendency of cardiovascular patient from formal help source and again more importantly, those physician working in cardiac clinic should screen patient for depression and link to psychiatric service.

## Abbreviations

AHSQ: Actual Help Seeking Questionnaire
CAD: coronary artery disease
CVD: cardiovascular disease
DALYs: disability adjusted life years
DDM: diabetes related heart disease
ETB: Ethiopian Birr
GP: General Practitioner
HF: heart failure
HIV/AIDS: human Immune virus/acquired immune deficiency syndrome
HHD: hypertensive related heart disease
IHD: ischemic heart disease
JU: Jimma University
JUTH: Jimma University teaching hospital
LMIC: low- and middle-income countries
MD: major depression
OSS-3: Oslo social support scale -3
QOL: quality of Life
PHQ-9: Patient Health Questionnaire
SPSS: statically package social science
WHO: World Health Organization
